# The potential of learning with (and not from) artificial intelligence in education

**DOI:** 10.3389/frai.2022.903051

**Published:** 2022-09-13

**Authors:** Tanya Chichekian, Bérenger Benteux

**Affiliations:** Faculty of Education, Université de Sherbrooke, Longueuil, QC, Canada

**Keywords:** artificial intelligence, intelligent tutoring system, learning, performance, education

## Abstract

AI-powered technologies are increasingly being developed for educational purposes to contribute to students' academic performance and overall better learning outcomes. This exploratory review uses the PRISMA approach to describe how the effectiveness of AI-driven technologies is being measured, as well as the roles attributed to teachers, and the theoretical and practical contributions derived from the interventions. Findings from 48 articles highlighted that learning outcomes were more aligned with the optimization of AI systems, mostly nested in a computer science perspective, and did not consider teachers in an active role in the research. Most studies proved to be atheoretical and practical contributions were limited to enhancing the design of the AI system. We discuss the importance of developing complementary research designs for AI-powered tools to be integrated optimally into education.

## Introduction

In the last decade, there has been a surge of educational research about how to effectively integrate technology in classrooms, with a focus on providing digital experiences that improve students' academic performance. With recent movements regarding the use of artificial intelligence (AI) as the leading medium by which we engage students in scholarly tasks (Roll and Wylie, 2016), rethinking how to design such technology is imperative if the intent is to facilitate the learning processes that lead to the achievement of learning objectives and, ultimately, to optimal functioning in education.

AI is defined by Popenici and Kerr (2017) as “computing systems that can engage in human-like processes such as learning, adapting, synthesizing, self-correction and use of data for complex processing tasks” (p. 2). These systems, which are displayed in various forms ranging from Internet search engines to smartphone applications, are shaping new teaching and learning educational contexts (Pedró et al., 2019). Generally, educational technologies driven by AI-powered algorithms are referred to as Intelligent Tutoring System (ITS) and try to replicate human tutor interactions (VanLehn, 2011) through a pedagogical agent by providing timely feedback and guidance to students (Kay, 2012). However, while ITSs have made advancements in the field of education, specifically in online environments or in computer labs, it remains unclear as to how their effectiveness can be interpreted or translated regarding the quality of students' learning outcomes (Pedró et al., 2019). This is partly due to the minimal evidence and support for wider adoption of the term “learning” on the part of the AIED community, and even less attention attributed to developing a well-defined role for teachers implementing these technologies. The latter is reflected in AIED research being published mostly in specialized journals and conference proceedings, which rarely become visible to educational researchers and only include limited educational perspectives in line with these technological developments (Zawacki-Richter et al., 2019). Although there is some strength with AIED and ITS conferences providing opportunities for the cross-fertilization of approaches, techniques, and ideas stemming from multidisciplinary research fields, it also creates a massive challenge for the AIED community in terms of communicating successfully both within the field and beyond, particularly with key actors in the wider education community. Tuomi (2018) also stated that there is a high chance that the way current AIED systems are being designed and developed is far from the learning outcomes learning scientists and teachers are expecting from these tools, especially if most AIED research has a weak connection to theoretical and pedagogical perspectives and is more aligned as a system of inputs and outputs.

The current exploratory review's purpose was to interpret findings from AIED research using a pedagogical perspective. Such an investigation is important for the following reasons. First, practical implications need to be considered in the field of education if certain conditions are to be fulfilled and resources reinvested to facilitate pedagogical activities. This is essential when examining whether the AIED field has the potential to be impactful in authentic situations. Such practical implications are also helpful for decision-makers in determining adequate funding policies for AIED research and projects. Second, given the growing trend in the number of publications in AIED (Chen et al., 2020a,b), interest in the field is in expansion. As such, the publication sources and conference venues play a major role in helping the educational community identify relevant information and findings that can be reflected in the progress and advancement of this field in educational settings. Third, it guides individuals from different disciplines to be exposed, to understand, and analyze the use of AI-driven technologies from multiple perspectives and thus visualize innovative ways of adapting them for educational purposes.

Accordingly, this review aims to answer the following research questions:

- RQ1: How is the effectiveness of an ITS measured in AIED research?
- RQ2: To what extent does AIED research contribute to the field of education?

This review contributes to the research field by enabling the educational community to understand the relationship between students' learning gains and the role of the ITS. Furthermore, it provides a knowledge base from which educational and computer science researchers can extrapolate to build, design, and collaborate on projects that are suitable for scaling up.

## State-of-the-art in AIED research

According to a meta-analysis conducted by Ma et al. (2014), an ITS is composed of four elements: (1) an interface that communicates with the learner by presenting information, asking questions, assigning learning tasks, providing feedback, and answering questions posed by students, (2) a domain model that represents the knowledge intended for the student to learn, (3) a student model that represents relevant aspects of the student's knowledge or psychological state determined by the student's responses to questions or other interactions with the interface, and (4) a tutoring model that adapts instructional strategies based on the needs of the learners. On a cognitive level, ITSs have facilitated students' learning processes during homework and practice exercises in the absence of a teacher or a tutor (VanLehn, 2011). Given that their use has often led to significantly higher achievement outcomes compared to other modes of instruction (Ma et al., 2014), they are often considered one of the resources in educators' toolboxes (Steenbergen-Hu and Cooper, 2013). In terms of supporting students' learning processes, ITSs seem to be most impactful on metacognitive strategies by prompting students to apply self-regulation skills and monitor their progress when learning (Bouchet et al., 2016). For example, Verginis et al. (2011) showed that the use of an open-learner model guided previously disengaged online students toward re-engagement and, ultimately, to improved post-test performance. Similarly, Arroyo et al. (2014) provided evidence of the positive impact that learning companions had on the improvement of low-achieving students' affective states and their motivation. It seems ITSs occupy an important and complementary place in learning and as a supplement to teachers' instruction.

### Impact on learning

In the last couple of years, numerous studies have started demonstrating how the use and impact of digital technologies and ITSs are directly related to the extent to which the technology itself is responsible for the observed increases in students' academic performance. Results from a meta-analysis (Schroeder et al., 2013) showed how pedagogical agents (PAs) had a small but significant positive effect on learning (*g* = 0.19, *p* < 0.05) among K-12 students compared to those who did not interact with PAs. Their learning gains were proportionally higher compared to collaborative interactions with other types of traditional, closed-ended, and teacher-led interactions or with non-ITS computer-based instruction (Harley et al., 2018). Arroyo et al. (2014) also demonstrated how students in these types of collaborative activities not only displayed an increase in learning gains but also passed standardized tests more frequently (92%) compared to a control group (79%) or to students who did not interact with any tutor (76%).

According to Steenbergen-Hu and Cooper (2013, 2014), certain variables have also been tested for moderating the significance of the effectiveness of an ITS. These include comparison conditions (e.g., traditional classroom instruction), type of ITS, subject matter, year of study, teacher involvement, assessment type, schooling level, length of the intervention, degree of implementation, students' prior knowledge, sample size, research design, self-regulation skills, and academic motivation. Specifically, ITSs produced the most significant impact depending on: (1) the year of study, (2) teacher involvement, and (3) the use of embedded assessments. It was rarely reported how process variables might help to explain observed effects or a lack thereof (Winne and Baker, 2013). Ma et al. (2014) indicated that whenever a process variable was reported in a study, it was often only meaningful in the context of the learning task. Therefore, when referring to the outperformance of an ITS compared to other methods of computer-based instruction, the effect at the level of computer-student interaction was rarely considered. Nevertheless, the use of ITSs to increase academic achievement was significant regardless of the context in which it was used. However, despite its effectiveness as a learning tool, the emergence and rapid growth of technology in education have resulted in a rushed deployment with not enough time to analyze how learning should be measured with the assistance of AI nor the extent to which teachers should implement these AI-driven learning experiences in the curriculum (Pedró et al., 2019).

### AI-driven learning experiences

Research and development on AIEd is still a young field in which the advancement of knowledge has the potential to make significant contributions to the learning sciences. Steenbergen-Hu and Cooper (2014) suggested various pedagogical hypotheses to move in such a direction such as experimentally adjusting the type of instruction and the frequency of feedback to optimize instruction and ITS equitably and meet the needs of different learners. Examples of such design strategies have thus far resided in the Computer-Human Interaction field. For example, Positive Technologies (Riva et al., 2012) applied templates from positive psychology to improve the technology's affective quality as well as promote students' engagement and connectedness with the content. Hassenzahl (2010) proposed an experiential approach for design to explore what matters to humans, what is needed to make technology more meaningful, and how to uncover “experience patterns” in human activities. Similarly, Positive Design (Desmet and Pohlmeyer, 2013), a framework for wellbeing, focused on how the design of any artifact or product might foster flourishing. Finally, as part of Positive Computing, Calvo and Peters (2014) provided leverage for a design supportive of wellbeing as well as its determinants. Although each of these frameworks provides some version of the core elements that are foundational to the learning sciences, these models remain at a distance from the field of education. Many articles about educational technology remain atheoretical (Hew et al., 2019) and lack focus on pedagogical perspectives (Bartolomé et al., 2018). To better understand, empirically evaluate, and design learning experiences about the impact of AI-driven technologies on students' academic success, as well as on certain psychological aspects that play a role in the learning process such as their motivation, we need to anchor them in conceptual or theoretical frameworks that take origin at the intersections of education, psychology, and computer science. One example is the Motivation, Engagement, and Thriving in User Experience model that was based on the self-determination theory (SDT, Deci and Ryans, 2002) to assess psychological needs in five different but interdependent contexts: at the point of technology adoption, during interaction with the interface, as a result of engagement with technology-specific tasks, as part of the technology-supported behavior, and as part of an individual's life overall. In addition to predicting the impact on motivation and sustained engagement with technology, the SDT can also serve as a basis to measure educational or other domain-specific outcomes, thus making it an ideal framework on which to build an understanding of common goals within technology projects.

## Research method

We searched the literature in the ERIC, PsycINFO, and Education Source databases as they contained the most publications regarding educational research. We used a combination of terms from the AIED and education fields such as “artificial intelligence,” “intelligent tutoring systems,” “natural language processing,” “student^*^,” and “learn^*^”. Additionally, we included synonyms found in the search databases' thesaurus that related to the term impact such as “impact^*^,” “effect^*^,” “outcome^*^,” “consequence^*^,” and “eval^*^”. More specifically, we searched the mentioned databases with the keywords “artificial intelligence” or “intelligent tutor^*^ systems” in the topic, then we used the connector “AND” to combine these results with the keywords “student^*^” or “learn^*^” and in a third step we combined these results, using the connector “AND”, with our keywords about impacts, namely “impact^*^” or “effect^*^” or “outcome^*^” or “consequence^*^” or “eval^*^”. This search resulted in a total of 479 articles (386 articles in ERIC, 79 in PsycINFO, and 32 in Education Source) which we imported to Zotero, a reference management system.

To begin the screening process, we first checked for duplicates which resulted in the deletion of 41 articles. We then scanned the remaining articles to decide if they met the following inclusion criteria:

- Evaluated the effects of AI on learning;
- Published in peer-reviewed journals;
- Took place between 2009 and 2019;
- Published in English.

**Figure 1:**
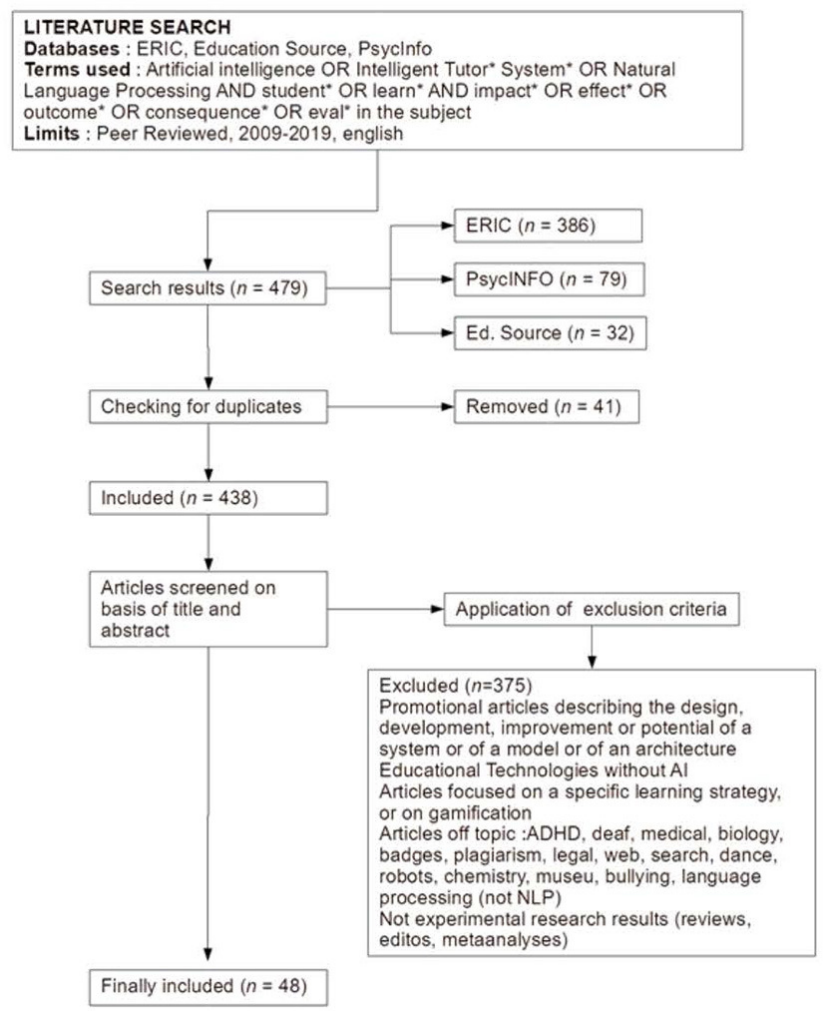
Flowchart of data selection.

A study needed to meet all our criteria to be included in the review. After deleting duplicates and applying these selection criteria to the remaining 438 publications, we narrowed down the review to 48 articles (see Figure 1). The most common reasons for which studies did not qualify for inclusion were that they focused primarily on the description of the design or development of a system, they addressed non-AI-powered educational technologies, and they evaluated systems' (not learner') outcomes. We coded and analyzed studies based on the following elements: the workplace and departments where the authors of the selected articles worked, theoretical framework, teachers' attributed role in the study, learning outcomes, as well as theoretical and practical contributions. In the following section, we present the current achievements in AIED followed by a summary of the findings from this literature search.

## Findings, analysis, and discussion

### The effectiveness of an ITS as measured in AIED research

Findings (see Supplementary Table 1) indicated that the effectiveness of an ITS was assessed by measuring students' learning gains, either as a difference between a pre and post-test [*n* =15 (31%)], as a perception of student learning [*n* = 19 (40%)], as a level of interaction with a learner during an activity, *n* = 6 (13%), or through standardized measurements such as national tests [*n* = 5 (10%)] or academic performance [*n* = 8 (17%)]. These results are in line with other meta-analytic reviews such as those from Kulik and Fletcher (2016) demonstrating the effectiveness of ITSs as instructional tools. Compared to students in conventional classes, those who received intelligent tutoring outperformed the others 92% of the time and this improved performance was significant enough 78% of the time. Moreover, the effectiveness of ITSs at times even surpassed other forms of computer or human tutoring.

These results are not surprising given that three meta-analytic reviews (Ma et al., 2014; Steenbergen-Hu and Cooper, 2014; Kulik, 2015) had also revealed the effectiveness of the ITS-related learning to be reflective of improved test score measurements. Our findings also concur with that of Guo et al. (2021) who advocated that despite high-level research in AIED, many were repetitive with few innovative breakthroughs in recent years. From an educational stance, this implies that the effects and functions that ITSs are seeking to achieve remain limited and, consequently, void of guidelines emanating from more robust theoretical frameworks nested in the learning sciences.

### AIED's contribution to the field of education

To examine the theoretical contributions of the selected studies to the AIED research community, we first noted the industry in which the authors worked. Of the 169 authors of the 48 articles, 78% (*n* = 132) were professors in a university department: 15% (*n* = 26) from education, 4% (*n* = 7) from educational psychology, 32% (*n* = 54) computer science, 12% (*n* = 20) from engineering, 11% (*n* = 18) from psychology, and 4% (*n* = 7) from other departments. In addition, 12% (*n* = 20) of the authors worked in a university, but not as a professor, and 10% (*n* = 17) were not from academia. Next, our review revealed that only *n* = 10 (6%) studies referred to a theoretical framework that supported their research study of which *n* = 6 originated from the field of educational psychology and *n* = 4 from pedagogy. Finally, of the 10 articles referring to a theoretical framework, *n* = 6 (4%) also mentioned a theoretical contribution of their study findings to the field (*n* = 6 in educational psychology, *n* = 4 in pedagogy, and *n* = 1 in cognitive psychology), either as a replication of previous research (*n* = 4) or as an extension to current knowledge (*n* = 2). In terms of the practical contributions associated with each study, *n* = 39 (81%) studies targeted the optimization of the ITS's performance and *n* = 8 contributed to improving the design of an ITS. Only one article had no practical contribution as its goal was to make a theoretical contribution and confirm previous research in the field. The significant gap between the theoretical and practical contributions is aligned with the focus on online learning, especially during the pandemic with an exponential increase in the utilization of AI-powered educational technology tools (Chaudhry and Kazim, 2022). A lot more work needs to be done on outlining the theoretical contributions of AIED as we move forward with a growing number of AI-powered educational technology that has the potential of producing a long-lasting educational and psychological impact on learners and teachers equally.

Overall, these findings demonstrate that the field of AIED seems to be targeting outcomes related more to the optimization of AI systems compared to the quality of learning itself. Moreover, most of the studies we reviewed only evaluated the impacts of these AI-powered technologies from a computer science perspective. Rarely were the studies framed and conceived as research contributing to a theoretical question about the relationship between ITS and learning outcomes. This is in line with past findings revealing very little evidence for the advancement of a pedagogical perspective and learning theories in AIED research (Bartolomé et al., 2018). To develop a complementary research design embedded within an educational framework (Pedro, 2019), integrating interdisciplinary perspectives about how to use AI for learning in educational settings is a future avenue worth exploring. It seems there is still substantial room to adopt a more participatory approach with educators if the field of AIED is to produce a critical reflection regarding the pedagogical and ethical implications of implementing AI applications in classrooms and, more importantly, to contribute to the advancement of learning theories with an appropriate and aligned conceptual or theoretical framework.

## Conclusion

This exploratory review highlighted that the purpose of most educational research with AI-driven technologies was to demonstrate the effectiveness of an ITS by measuring students' academic performance. Although recent studies have shown how these technologies also contribute to overall better learning outcomes among students (Laanpere et al., 2014; Luckin et al., 2016), very few have been implemented as applications in classrooms. To capitalize on students' optimal learning (Ryan and Deci, 2017), in addition to academic performance, positive learning experiences need to be designed that consider students' interactions with AI, including the maintenance of a certain level of motivation and engagement (Niemic and Ryan, 2009; Peters et al., 2018), as well as a well-defined role for the classroom teacher. With a more proactive role assigned to classroom teachers involved in collaborative research, the need to integrate their perspectives in AI-driven educational technological developments would be integral in understanding student learning in a sociotechnical approach. Combining a technical system and a classroom culture requires different levels of adaptability given that an ITS needs to be designed and integrated into both the students' learning and the teacher's instruction. Perhaps the next challenge for the AIED community is to determine a more equitable division of labor between the roles of the teacher and of the intelligent tutoring system, both of which support students with instructions, tasks, and feedback. Self-adaptive systems could enable a transformation in educational practice (Tuomi, 2018), but the challenge remains in deciding whether the intelligent tutoring system or the teaching activities should be re-designed and re-aligned with the other.

## Author contributions

TC contributed to conception and design of the study and the other sections of the manuscript. BB organized the database, performed the search, selection of studies for the review, and wrote the results. All authors contributed to manuscript revision.

## Conflict of interest

The authors declare that the research was conducted in the absence of any commercial or financial relationships that could be construed as a potential conflict of interest.

## Publisher's note

All claims expressed in this article are solely those of the authors and do not necessarily represent those of their affiliated organizations, or those of the publisher, the editors and the reviewers. Any product that may be evaluated in this article, or claim that may be made by its manufacturer, is not guaranteed or endorsed by the publisher.
