# Supplementary material for: The potential of learning with (and not from) artificial intelligence in education
Source: Front Artif Intell. 2022 Sep 13;5:903051. doi: 10.3389/frai.2022.903051 (PMC9513244; doi:10.3389/frai.2022.903051)
Supplement: Supplementary file 1 [file Table_1.DOCX]

**Table of results from selected studies**

| Reference | Effectiveness of ITS | Theoretical Contribution | Practical contribution | Teachers' role |
| --- | --- | --- | --- | --- |
| (Allen et al., 2016) | STP |  | SE | None |
| (Aravind & Refugio, 2019) | ETvsCT |  | SE | TGC |
| (Beal et al., 2010) | LGPrePost |  | SE | None |
| (Behrend & Thompson, 2012) | ETvsCT | X | DA | None |
| (Beigman Klebanov et al., 2017) | ETvsCT |  | SE | None |
| (Benotti et al., 2018) | LIwS, SPoL |  | SE | TGC |
| (Broisin et al., 2017) | LIwS, AP |  | SE | CAM |
| (Chin et al., 2010) | ETvsCT |  | SE | TGC |
| (Chu et al., 2014) | LGPrePost |  | SE | CAM |
| (Cung et al., 2019) | AP |  | DA | TGC |
| (Donnelly et al., 2015) | ETvsCT | X |  | TGC, CD, Participant |
| (Enright & Quinlan, 2010) | ETvsCT |  | SE | None |
| (Gaudioso et al., 2009) | LIwS, SPoL, TPoL, AP |  | SE | Participant |
| (Grimes & Warschauer, 2010) | SPoL, TPoL |  | SE | AS |
| (Harley et al., 2018) | LGPrePost | X | SE | None |
| (Holmes et al., 2018) | ETvsCT |  | DA | None |
| (Hsieh et al., 2012) | LGPrePost, SPoL |  | SE | None |
| (Huang et al., 2011) | ETvsCT |  | SE | CD |
| (Hull & du Boulay, 2015) | LGPrePost |  | DA | None |
| (Hwang et al., 2016) | SPoL, AP |  | SE | TGC |
| (Jena, 2018) | LGPrePost |  | SE | None |
| (Ji et al., 2018) | STP |  | SE | None |
| (Johnson, 2007) | ETvsCT |  | SE | None |
| (Karaci et al., 2018) | LGPrePost |  | SE | CAM |
| (Ketamo & Suominen, 2010) | LGPrePost |  | SE | AS |
| (Liou et al., 2012) | SPoL |  | SE | None |
| (Liu & Koedinger, 2017) | LGPrePost |  | SE | None |
| (Méndez & González, 2010) | SPoL, AP |  | SE | TGC, CD |
| (Mostow et al., 2013) | LGPrePost |  | SE | None |
| (Nabiyev et al., 2013) | SPoL, TPoL, AP |  | SE | Participant |
| (Nye et al., 2018) | LGPrePost, SPoL |  | DA | TGC |
| (Roschelle et al., 2016) | AP |  | SE | TGC |
| (Segedy et al., 2013) | LGPrePost |  | DA | TGC |
| (Serrano et al., 2018) | STP |  | SE | None |
| (Su et al., 2011) | SPoL, AP |  | SE | TGC, CAM |
| (Tärning et al., 2019) | LIwS, SPoL | X | DA | None |
| (Theodoridou, 2011) | ETvsCT |  | SE | None |
| (Thompson & McGill, 2017) | SPoL |  | SE | None |
| (VanLehn et al., 2017) | ETvsCT |  | SE | None |
| (Verdú et al., 2017) | LIwS, SPoL |  | SE | CAM |
| (Verginis et al., 2011) | LIwS, SPoL |  | SE | TGC |
| (Ward et al., 2013) | LGPrePost |  | SE | None |
| (K. Wijekumar et al., 2014) | STP | X | SE | TGC, CD |
| (K. Wijekumar et al., 2017) | STP | X | SE | TGC |
| (Zapata‐Rivera et al., 2009) | SPoL |  | SE | AS, CRD |
| (Zhang et al., 2009) | LGPrePost |  | SE | None |
| (Zhang & VanLehn, 2017) | ETvsCT |  | DA | None |
| (Zhao et al., 2012) | LGPrePost, SPoL |  | SE | CAM |

*Note.*

| LGPrePost = Learning gains as pre-post tests (n =15) | DA = design advices (n = 8) |
| --- | --- |
| LIwS = Learners interactions with the system during an activity (n = 6) | AS = Assist students with ITS (n = 3) |
| SPoL = Students' perceptions of learning (n =16) | TGC = Teach in control group (n =14) |
| TPoL = Teachers' perceptions of learning (n = 3) | CD = Collect data (n = 4) |
| EXvsCT = exp vs control (n = 12) | CAM = Consulted on assessment measures (n = 6) |
| AP = Academic performance (n = 8) | CRD = Consulted on research design (n = 1) |
| STP = Performance on standardized tests (n = 5) | Participant = Surveyed as participants (n = 3) |
| SE = efficiency of the system (n = 39) | No teacher role = None (n = 23) |

**List of references in the table**

Allen, L. K., Snow, E. L., and McNamara, D. S. (2016). The narrative waltz: The role of flexibility in writing proficiency. *Journal of Educational Psychology* 108, 911–924. doi:[10.1037/edu0000109](https://doi.org/10.1037/edu0000109).

Aravind, V. R., and Refugio, C. (2019). Efficient Learning with Intelligent Tutoring across Cultures. *World Journal on Educational Technology: Current Issues* 11, 30–37.

Beal, C. R., Arroyo, I. M., Cohen, P. R., and Woolf, B. P. (2010). Evaluation of AnimalWatch: An intelligent tutoring system for arithmetic and fractions. *Journal of Interactive Online Learning* 9, 64–77.

Behrend, T. S., and Thompson, L. F. (2012). Using animated agents in learner-controlled training: the effects of design control. *International Journal of Training and Development* 16, 263–283. doi:[10.1111/j.1468-2419.2012.00413.x](https://doi.org/10.1111/j.1468-2419.2012.00413.x).

Beigman Klebanov, B., Burstein, J., Harackiewicz, J. M., Priniski, S. J., and Mulholland, M. (2017). Reflective Writing About the Utility Value of Science as a Tool for Increasing STEM Motivation and Retention – Can AI Help Scale Up? *Int J Artif Intell Educ* 27, 791–818. doi:[10.1007/s40593-017-0141-4](https://doi.org/10.1007/s40593-017-0141-4).

Benotti, L., Martnez, M. C., and Schapachnik, F. (2018). A Tool for Introducing Computer Science with Automatic Formative Assessment. *IEEE Transactions on Learning Technologies* 11, 179–192. doi:[10.1109/TLT.2017.2682084](https://doi.org/10.1109/TLT.2017.2682084).

Broisin, J., Venant, R., and Vidal, P. (2017). Lab4CE: a Remote Laboratory for Computer Education. *Int J Artif Intell Educ* 27, 154–180. doi:[10.1007/s40593-015-0079-3](https://doi.org/10.1007/s40593-015-0079-3).

Chin, D. B., Dohmen, I. M., Cheng, B. H., Oppezzo, M. A., Chase, C. C., and Schwartz, D. L. (2010). Preparing students for future learning with Teachable Agents. *Education Tech Research Dev* 58, 649–669. doi:[10.1007/s11423-010-9154-5](https://doi.org/10.1007/s11423-010-9154-5).

Chu, Y.-S., Yang, H.-C., Tseng, S.-S., and Yang, C.-C. (2014). Implementation of a Model-Tracing-Based Learning Diagnosis System to Promote Elementary Students’ Learning in Mathematics. *Journal of Educational Technology & Society* 17, 347–357.

Cung, B., Xu, D., Eichhorn, S., and Warschauer, M. (2019). Getting Academically Underprepared Students Ready through College Developmental Education: Does the Course Delivery Format Matter? *American Journal of Distance Education* 33, 178–194. doi:[10.1080/08923647.2019.1582404](https://doi.org/10.1080/08923647.2019.1582404).

Donnelly, D. F., Vitale, J. M., and Linn, M. C. (2015). Automated Guidance for Thermodynamics Essays: Critiquing Versus Revisiting. *J Sci Educ Technol* 24, 861–874. doi:[10.1007/s10956-015-9569-1](https://doi.org/10.1007/s10956-015-9569-1).

Enright, M. K., and Quinlan, T. (2010). Complementing human judgment of essays written by English language learners with e-rater® scoring. *Language Testing* 27, 317–334. doi:[10.1177/0265532210363144](https://doi.org/10.1177/0265532210363144).

Gaudioso, E., Hernandez-del-Olmo, F., and Montero, M. (2009). Enhancing E-Learning Through Teacher Support: Two Experiences. *IEEE Transactions on Education* 52, 109–115. doi:[10.1109/TE.2008.919810](https://doi.org/10.1109/TE.2008.919810).

Grimes, D., and Warschauer, M. (2010). Utility in a Fallible Tool: A Multi-Site Case Study of Automated Writing Evaluation. *The Journal of Technology, Learning and Assessment* 8. Available at: <https://ejournals.bc.edu/index.php/jtla/article/view/1625> [Accessed March 18, 2022].

Harley, J. M., Taub, M., Azevedo, R., and Bouchet, F. (2018). Let’s Set Up Some Subgoals: Understanding Human-Pedagogical Agent Collaborations and Their Implications for Learning and Prompt and Feedback Compliance. *IEEE Transactions on Learning Technologies* 11, 54–66. doi:[10.1109/TLT.2017.2756629](https://doi.org/10.1109/TLT.2017.2756629).

Holmes, M., Latham, A., Crockett, K., and O’Shea, J. D. (2018). Near Real-Time Comprehension Classification with Artificial Neural Networks: Decoding e-Learner Non-Verbal Behavior. *IEEE Transactions on Learning Technologies* 11, 5–12. doi:[10.1109/TLT.2017.2754497](https://doi.org/10.1109/TLT.2017.2754497).

Hsieh, T.-C., Wang, T.-I., Su, C.-Y., and Lee, M.-C. (2012). A Fuzzy Logic-based Personalized Learning System for Supporting Adaptive English Learning. *Journal of Educational Technology & Society* 15, 273–288.

Huang, C.-J., Wang, Y.-W., Huang, T.-H., Chen, Y.-C., Chen, H.-M., and Chang, S.-C. (2011). Performance evaluation of an online argumentation learning assistance agent. *Computers & Education* 57, 1270–1280. doi:[10.1016/j.compedu.2011.01.013](https://doi.org/10.1016/j.compedu.2011.01.013).

Hull, A., and du Boulay, B. (2015). Motivational and metacognitive feedback in SQL-Tutor*. *Computer Science Education* 25, 238–256. doi:[10.1080/08993408.2015.1033143](https://doi.org/10.1080/08993408.2015.1033143).

Hwang, G.-H., Chen, B., and Huang, C.-W. (2016). Development and Effectiveness Analysis of a Personalized Ubiquitous Multi-Device Certification Tutoring System Based on Bloom’s Taxonomy of Educational Objectives. *Journal of Educational Technology & Society* 19, 223–236.

Jena, A. K. (2018). Predicting Learning Outputs and Retention through Neural Network Artificial Intelligence in Photosynthesis, Transpiration and Translocation. *Asia-Pacific Forum on Science Learning and Teaching* 19.

Ji, X. R., Beerwinkle, A., Wijekumar, K. (Kay), Lei, P., Malatesha Joshi, R., and Zhang, S. (2018). Using latent transition analysis to identify effects of an intelligent tutoring system on reading comprehension of seventh-grade students. *Read Writ* 31, 2095–2113. doi:[10.1007/s11145-018-9888-8](https://doi.org/10.1007/s11145-018-9888-8).

Johnson, W. (2007). “Serious use of a serious game for language learning,” in *Artificial Intelligence in Education: Building Technology Rich Learning Contexts that Work*, eds. R. Luckin, K. R. Koedinger, and J. E. Greer (Amsterdam, Netherlands: IOS Press), 67–75.

Karaci, A., Akyüz, H., Bi̇lgi̇ci̇, G., and Arici, N. (2018). Effects of Web-based Intelligent Tutoring Systems on Academic Achievement and Retention. *International Journal of Computer Applications* 181. doi:[10.5120/ijca2018917806](https://doi.org/10.5120/ijca2018917806).

Ketamo, H., and Suominen, M. (2010). Learning-by-Teaching in Educational Game: Educational Outcome, User Experience and Social Networks. *Journal of Interactive Learning Research* 21, 237–255.

Liou, H.-C., Yang, P.-C., and Chang, J. s. (2012). Language supports for journal abstract writing across disciplines. *Journal of Computer Assisted Learning* 28, 322–335. doi:[10.1111/j.1365-2729.2011.00446.x](https://doi.org/10.1111/j.1365-2729.2011.00446.x).

Liu, R., and Koedinger, K. R. (2017). Closing the Loop: Automated Data-Driven Cognitive Model Discoveries Lead to Improved Instruction and Learning Gains. *Journal of Educational Data Mining* 9, 25–41.

Méndez, J. A., and González, E. J. (2010). A reactive blended learning proposal for an introductory control engineering course. *Computers & Education* 54, 856–865. doi:[10.1016/j.compedu.2009.09.015](https://doi.org/10.1016/j.compedu.2009.09.015).

Mostow, J., Nelson-Taylor, J., and Beck, J. E. (2013). Computer-Guided Oral Reading versus Independent Practice: Comparison of Sustained Silent Reading to an Automated Reading Tutor That Listens. *Journal of Educational Computing Research* 49, 249–276. doi:[10.2190/EC.49.2.g](https://doi.org/10.2190/EC.49.2.g).

Nabiyev, V., Karal, H., Arslan, S., Erumit, A. K., and Cebi, A. (2013). An Artificial Intelligence-Based Distance Education System: Artimat. *Turkish Online Journal of Distance Education* 14, Article 4.

Nye, B. D., Pavlik, P. I., Windsor, A., Olney, A. M., Hajeer, M., and Hu, X. (2018). SKOPE-IT (Shareable Knowledge Objects as Portable Intelligent Tutors): overlaying natural language tutoring on an adaptive learning system for mathematics. *IJ STEM Ed* 5, 12. doi:[10.1186/s40594-018-0109-4](https://doi.org/10.1186/s40594-018-0109-4).

Roschelle, J., Feng, M., Murphy, R. F., and Mason, C. A. (2016). Online Mathematics Homework Increases Student Achievement. *AERA Open* 2, 2332858416673968. doi:[10.1177/2332858416673968](https://doi.org/10.1177/2332858416673968).

Segedy, J. R., Kinnebrew, J. S., and Biswas, G. (2013). The effect of contextualized conversational feedback in a complex open-ended learning environment. *Education Tech Research Dev* 61, 71–89. doi:[10.1007/s11423-012-9275-0](https://doi.org/10.1007/s11423-012-9275-0).

Serrano, M.-Á., Vidal-Abarca, E., and Ferrer, A. (2018). Teaching self-regulation strategies via an intelligent tutoring system (TuinLECweb): Effects for low-skilled comprehenders. *Journal of Computer Assisted Learning* 34, 515–525. doi:[10.1111/jcal.12256](https://doi.org/10.1111/jcal.12256).

Su, J.-M., Lin, H.-Y., Tseng, S.-S., and Lu, C.-J. (2011). OPASS: An Online Portfolio Assessment and Diagnosis Scheme to Support Web-Based Scientific Inquiry Experiments. *Turkish Online Journal of Educational Technology - TOJET* 10, 151–173.

Tärning, B., Silvervarg, A., Gulz, A., and Haake, M. (2019). Instructing a Teachable Agent with Low or High Self-Efficacy – Does Similarity Attract? *Int J Artif Intell Educ* 29, 89–121. doi:[10.1007/s40593-018-0167-2](https://doi.org/10.1007/s40593-018-0167-2).

Theodoridou, K. (2011). Learning Spanish with Laura: the effects of a pedagogical agent. *Educational Media International* 48, 335–351. doi:[10.1080/09523987.2011.632280](https://doi.org/10.1080/09523987.2011.632280).

Thompson, N., and McGill, T. J. (2017). Genetics with Jean: the design, development and evaluation of an affective tutoring system. *Education Tech Research Dev* 65, 279–299. doi:[10.1007/s11423-016-9470-5](https://doi.org/10.1007/s11423-016-9470-5).

VanLehn, K., Zhang, L., Burleson, W., Girard, S., and Hidago-Pontet, Y. (2017). Can a Non-Cognitive Learning Companion Increase the Effectiveness of a Meta-Cognitive Learning Strategy? *IEEE Transactions on Learning Technologies* 10, 277–289. doi:[10.1109/TLT.2016.2594775](https://doi.org/10.1109/TLT.2016.2594775).

Verdú, E., Regueras, L. M., Gal, E., de Castro, J. P., Verdú, M. J., and Kohen-Vacs, D. (2017). Integration of an intelligent tutoring system in a course of computer network design. *Education Tech Research Dev* 65, 653–677. doi:[10.1007/s11423-016-9503-0](https://doi.org/10.1007/s11423-016-9503-0).

Verginis, I., Gouli, E., Gogoulou, A., and Grigoriadou, M. (2011). Guiding Learners into Reengagement through the SCALE Environment: An Empirical Study. *IEEE Transactions on Learning Technologies* 4, 275–290. doi:[10.1109/TLT.2011.20](https://doi.org/10.1109/TLT.2011.20).

Ward, W., Cole, R., Bolaños, D., Buchenroth-Martin, C., Svirsky, E., and Weston, T. (2013). My science tutor: A conversational multimedia virtual tutor. *Journal of Educational Psychology* 105, 1115–1125. doi:[10.1037/a0031589](https://doi.org/10.1037/a0031589).

Wijekumar, K., Meyer, B. J. F., and Lei, P. (2017). Web-based text structure strategy instruction improves seventh graders’ content area reading comprehension. *Journal of Educational Psychology* 109, 741–760. doi:[10.1037/edu0000168](https://doi.org/10.1037/edu0000168).

Wijekumar, K., Meyer, B. J. F., Lei, P.-W., Lin, Y.-C., Johnson, L. A., Spielvogel, J. A., et al. (2014). Multisite Randomized Controlled Trial Examining Intelligent Tutoring of Structure Strategy for Fifth-Grade Readers. *Journal of Research on Educational Effectiveness* 7, 331–357. doi:[10.1080/19345747.2013.853333](https://doi.org/10.1080/19345747.2013.853333).

Zapata‐Rivera, D., VanWinkle, W., Doyle, B., Buteux, A., and Bauer, M. (2009). Combining learning and assessment in assessment‐based gaming environments: A case study from a New York City school. *Interactive Technology and Smart Education* 6, 173–188. doi:[10.1108/17415650911005384](https://doi.org/10.1108/17415650911005384).

Zhang, L., Gillies, M., Dhaliwal, K., Gower, A., Robertson, D., and Crabtree, B. (2009). E-Drama: Facilitating Online Role-Play Using an AI Actor and Emotionally Expressive Characters. *International Journal of Artificial Intelligence in Education* 19, 5–38.

Zhang, L., and VanLehn, K. (2017). Adaptively selecting biology questions generated from a semantic network. *Interactive Learning Environments* 25, 828–846. doi:[10.1080/10494820.2016.1190939](https://doi.org/10.1080/10494820.2016.1190939).

Zhao, G., Ailiya, and Shen, Z. (2012). Learning-by-Teaching: Designing Teachable Agents with Intrinsic Motivation. *Journal of Educational Technology & Society* 15, 62–74.
